# CTXφ Replication Depends on the Histone-Like HU Protein and the UvrD Helicase

**DOI:** 10.1371/journal.pgen.1005256

**Published:** 2015-05-20

**Authors:** Eriel Martínez, Evelyne Paly, François-Xavier Barre

**Affiliations:** Institute for Integrative Biology of the Cell (I2BC), Université Paris Saclay, CEA, CNRS, Université Paris Sud, Gif sur Yvette, France; Harvard University, UNITED STATES

## Abstract

The *Vibrio cholerae* bacterium is the agent of cholera. The capacity to produce the cholera toxin, which is responsible for the deadly diarrhea associated with cholera epidemics, is encoded in the genome of a filamentous phage, CTXφ. Rolling-circle replication (RCR) is central to the life cycle of CTXφ because amplification of the phage genome permits its efficient integration into the genome and its packaging into new viral particles. A single phage-encoded HUH endonuclease initiates RCR of the proto-typical filamentous phages of *enterobacteriaceae* by introducing a nick at a specific position of the double stranded DNA form of the phage genome. The rest of the process is driven by host factors that are either essential or crucial for the replication of the host genome, such as the Rep SF1 helicase. In contrast, we show here that the histone-like HU protein of *V*. *cholerae* is necessary for the introduction of a nick by the HUH endonuclease of CTXφ. We further show that CTXφ RCR depends on a SF1 helicase normally implicated in DNA repair, UvrD, rather than Rep. In addition to CTXφ, we show that VGJφ, a representative member of a second family of vibrio integrative filamentous phages, requires UvrD and HU for RCR while TLCφ, a satellite phage, depends on Rep and is independent from HU.

## Author Summary

One of the major strategies to prevent Cholera epidemics is the development of oral vaccines based on live attenuated *Vibrio cholerae* strains. The most promising vaccine strains have been obtained by deletion of the cholera toxin genes, which are harboured in the genome of an integrated phage, CTXϕ. However, they can re-acquire the cholera toxin genes when re-infected by CTXϕ or by hybrid phages between CTXϕ and other vibrio phages, which raised safety concerns about their use. Here, we developed a screening strategy to identify non-essential host factors implicated in CTXϕ replication. We show that the histone-like HU protein and the UvrD helicase are both absolutely required for its replication. We further show that they are essential for the replication of VGJϕ, a representative member of a family of phages that can form hybrids with CTXϕ. Accordingly, we demonstrate that the disruption of the two subunits of HU and/or of UvrD prevents infection of the *V*. *cholerae* by CTXϕ and VGJϕ. In addition, we show that it limits CTXϕ horizontal transmission. Taken together, these results indicate that HU^-^ and/or UvrD^-^ cells are promising candidates for the development of safer live attenuated cholera vaccine.

## Introduction

Cholera remains a major health problem in many part of the developing world, with an estimation of 2.8 million cases and 100 000 to 200 000 deaths each year [1]. The agent of the cholera, the *Vibrio cholerae* bacterium, is found in briny waters all over the world [2]. However, most *V*. *cholerae* strains are not pathogenic or only cause local outbreaks of gastroenteritis. Pathogenicity depends on the acquisition of several virulence factors, of which the cholera toxin (CT) and the toxin-coregulated pilus (TCP) are considered the most significant. CT causes a voluminous watery diarrhoea, which is responsible for the high rate of death associated with cholera and its epidemic propagation [3], while TCP is required for colonization of the small intestine [4]. The cholera toxin genes, *ctxAB*, are encoded in the genome of a lysogenic filamentous phage, CTXϕ [5]. The genomic characterization of *V*. *cholerae* epidemic strains suggested that several independent toxigenic conversion events occurred in the history of cholera [6–8], which motivated studies on the life cycle of CTXϕ.

**Fig 1:**
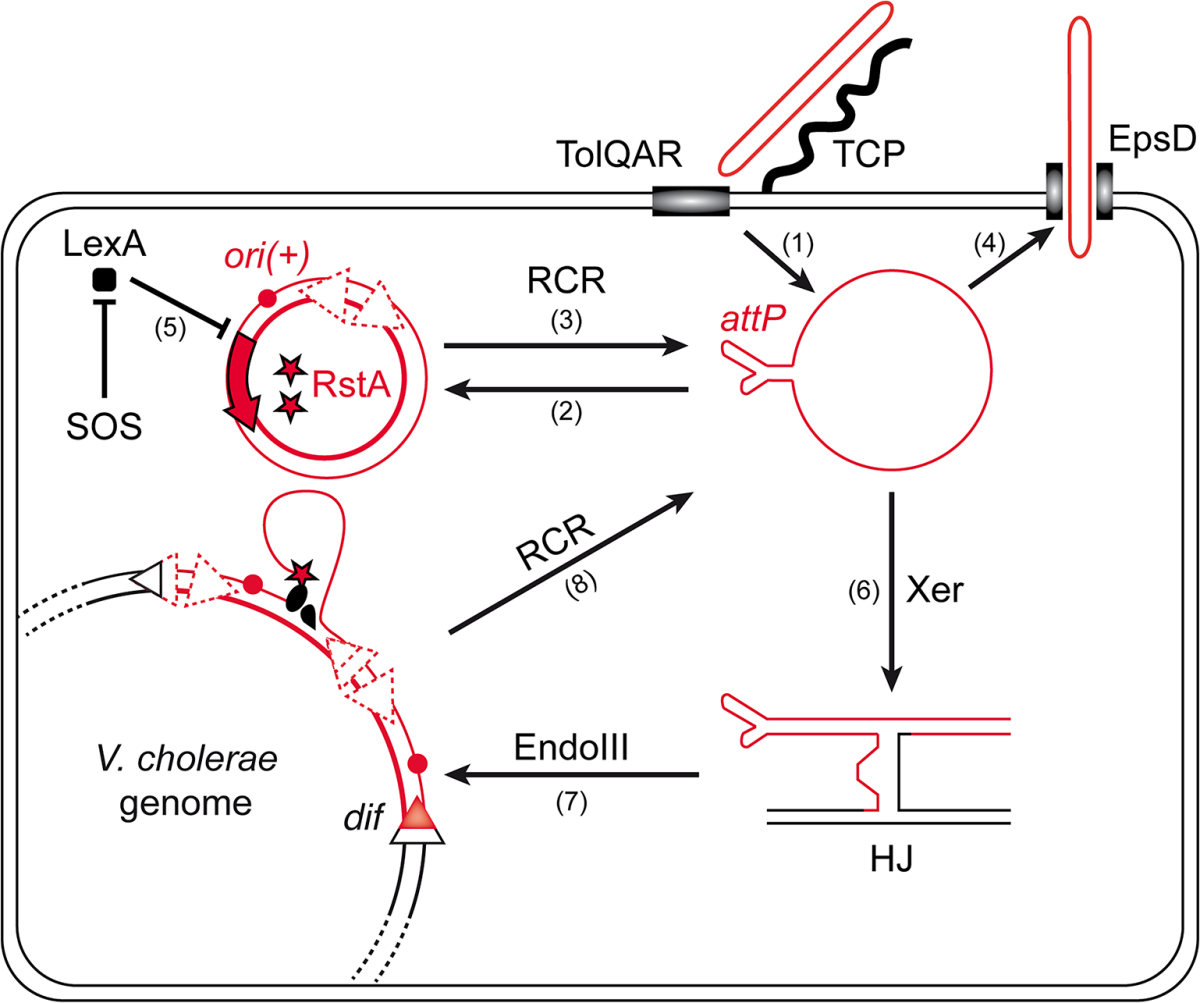
Rolling-Circle Replication is central to the life cycle of CTXϕ. Schematic diagram showing key steps in the life cycle of CTXϕ. CTXϕ infection requires the host-encoded toxin co-regulated pilus (TCP) and TolQRA proteins (1). After its release in the cytoplasm of its host, CTXϕ ssDNA is converted into a dsDNA by the host machineries (2). Rolling circle replication (RCR) of the phage depends on a single phage-encoded protein, RstA, and on the host machinery (3). New CTXϕ particle secretion depends on the host outer membrane protein EpsD (4). RstA production is under the control of the SOS response (5). Integration of CTXϕ depends on the host Xer machinery (6) and the accessory protein EndoIII (7). A process akin to RCR permits the production of free copies of CTXϕ ssDNA when the phage genome is integrated in tandem (8).

**Fig 2:**
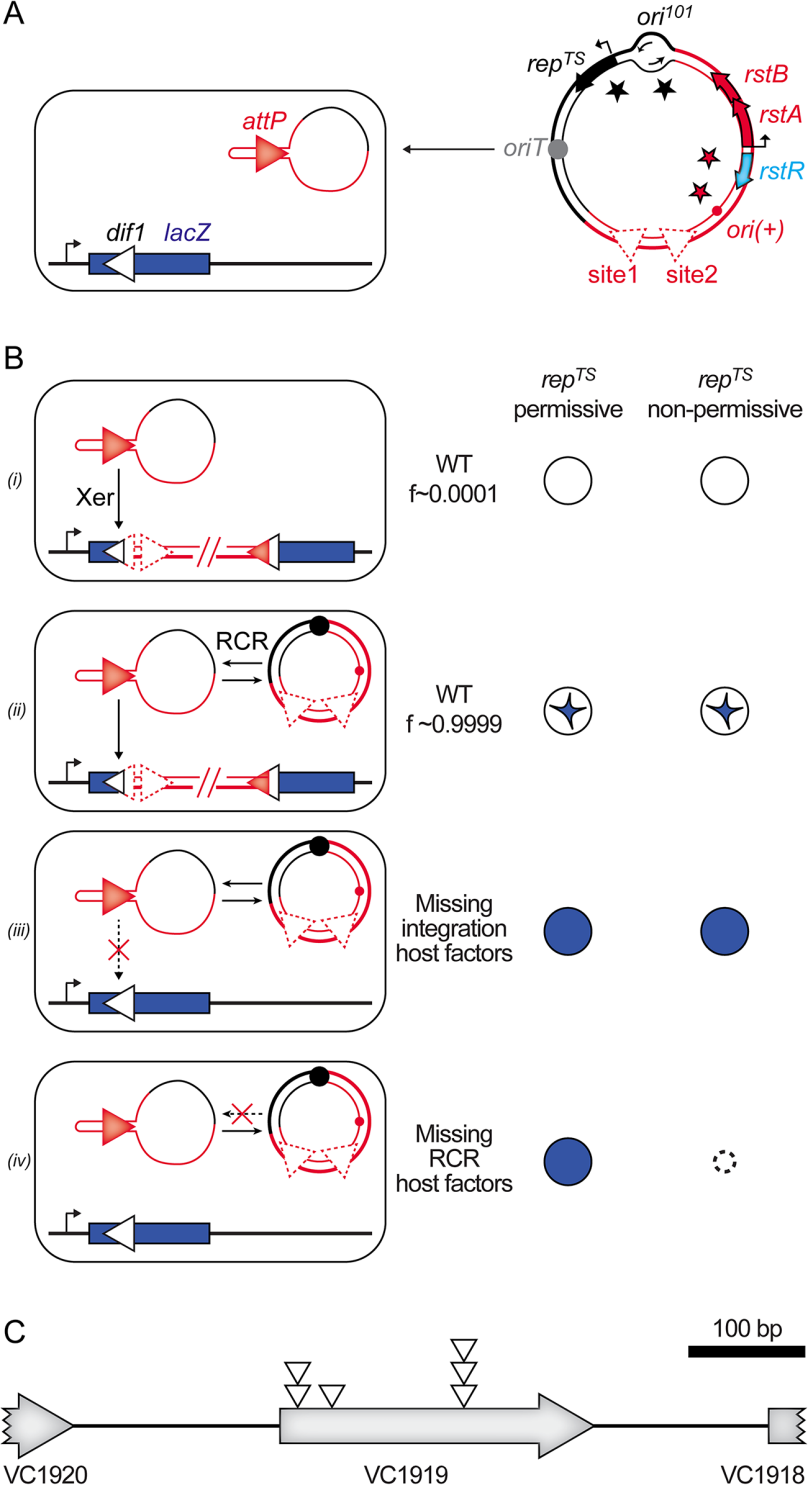
Screen for host factors implicated in CTXϕ replication. **(A)** Scheme of the conjugation of the pSC101-RS2 hybrid into a *lac*Z::*dif*1 reporter strain. **(B)** Schematic representation of the colonies obtained in different genetic backgrounds. *f*: relative frequency of formation of each type of depicted colony; (*i)*: colony formed upon direct integration; (*ii)*: colony obtained after RCR amplification of the phage DNA; (*iii)*: colony obtained when host factors implicated in integration are disrupted; (iv): colony obtained when host factors implicated in RCR are disrupted. **(C)** Scheme of the VC1919 region. Open triangles indicate the position of insertion of the transposon that impeded pSC101-RS2 RCR.

**Fig 3:**
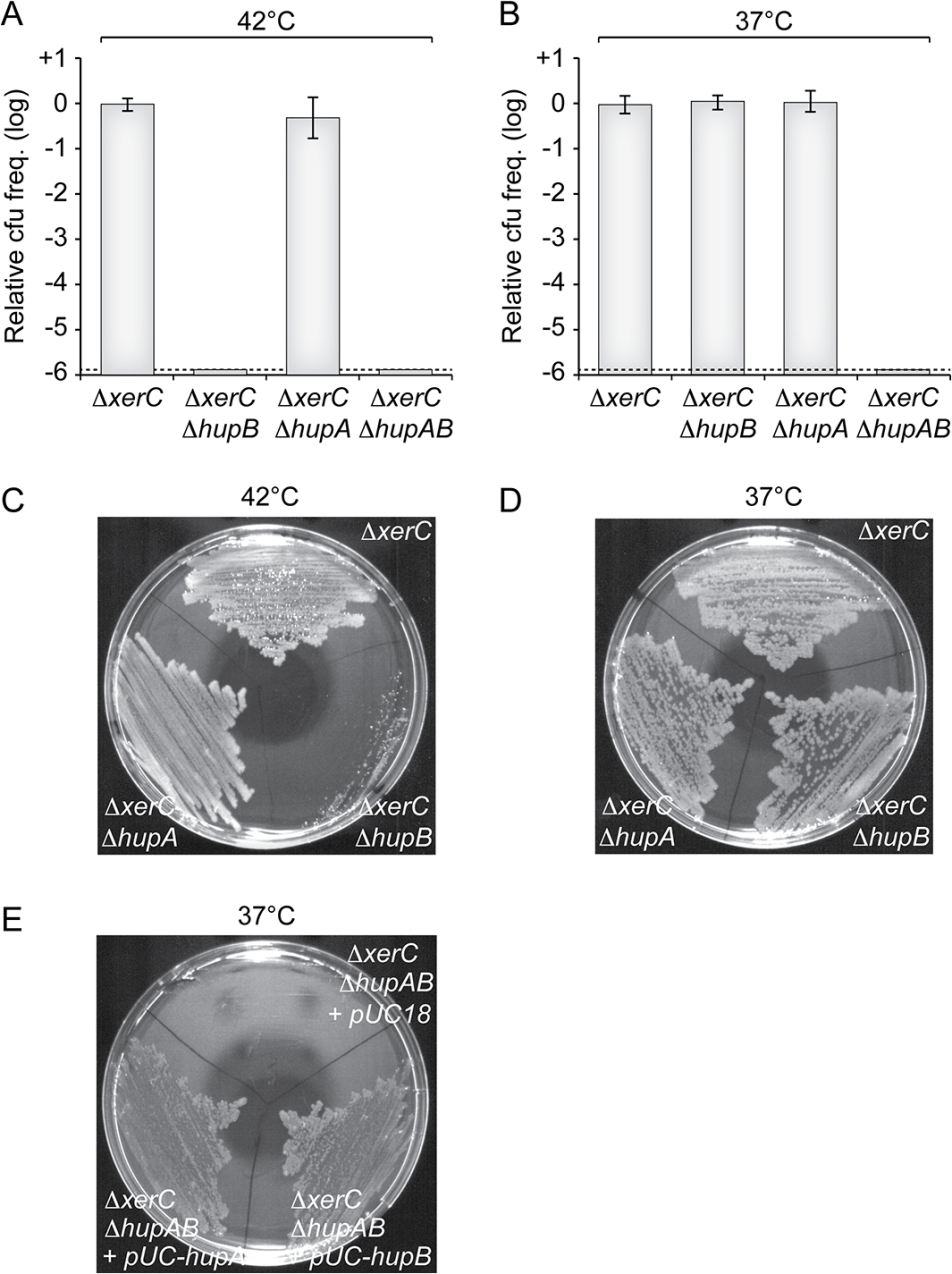
HU is essential for CTXϕ replication. **(A and B)** Relative colony-forming ability after conjugation with a replicative form of RS2 in the indicated strains. After 3 h of conjugation, serial dilutions were plated on chloramphenicol and incubated overnight at 42°C (A) and 37°C (B). Relative colony forming units (cfu) correspond to the ratio of the number of colonies obtained in the indicated strain over the mean number of colonies obtained in ∆*xerC* cells. Results are shown in a logarithmic scale and represent the mean and standard deviation of 3 independent experiments. The detection limit of the experiment is indicated by a dotted line. **(C and D)** Colonies obtained at 37°C were re-streaked on plate and incubated overnight at 42°C (C) and 37°C (D). **(E)** Complementation assay of Δ*hupAB* cells with a pUC18 vector carrying the *V*. *cholerae hupA* gene (pUC-*hupA*) *or hupB* gene (pUC-*hupB*). RS2 was conjugated into Δ*xerC* Δ*hupAB* + pUC18, Δ*xerC* Δ*hupAB* + pUC-*hupA* and Δ*xerC* Δ*hupAB* + pUC-*hupB*. The conjugants were then streaked on a plate supplemented with ampicillin and chloramphenicol. The plate was incubated overnight at 37°C.

**Fig 4:**
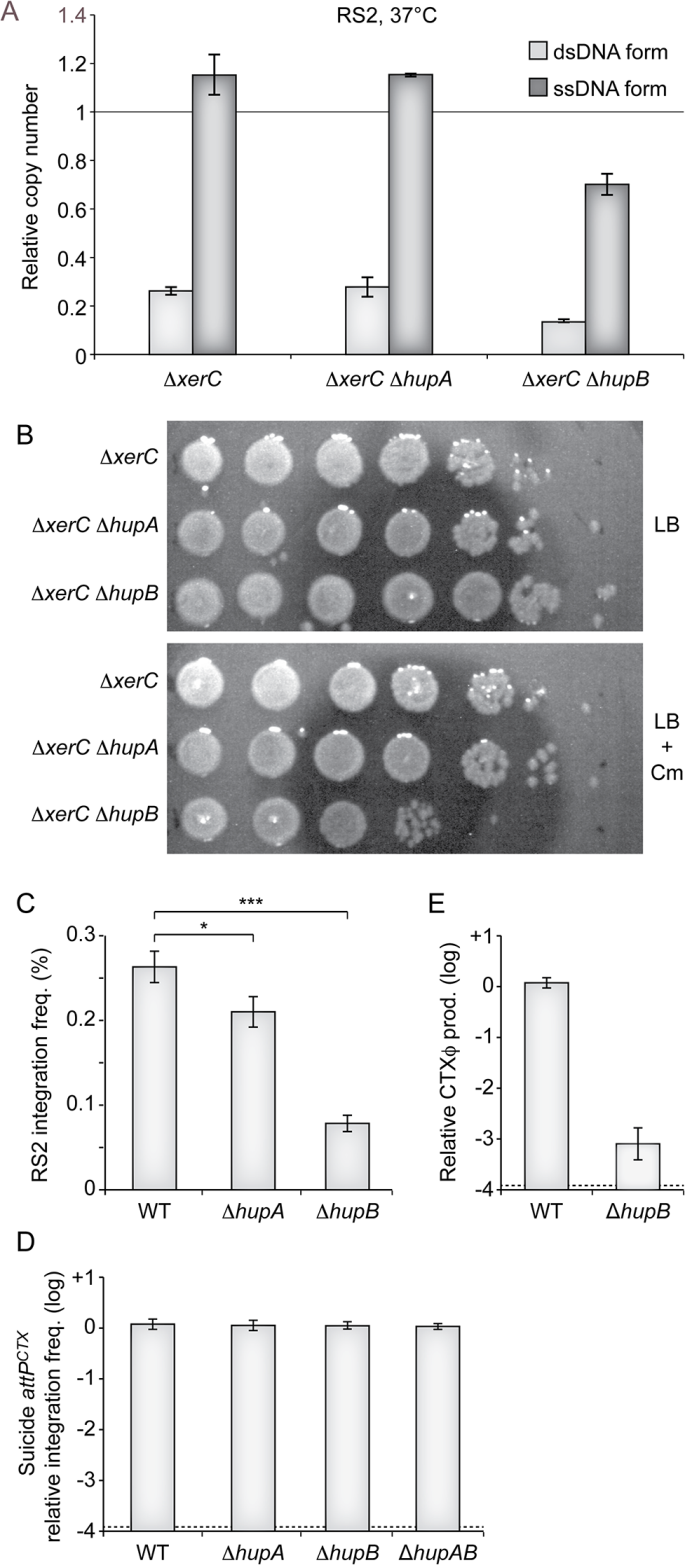
Impaired replication of CTXϕ in ∆*hupB* cells. **(A)** Q-PCR analysis of the number ssDNA and dsDNA copies of RS2 in the indicated strain*s*. The analysis was performed on the total DNA of cells that were grown under selection pressure at 30°C to an OD_600nm_ of 0.3. Data represent the mean of two independent experiments. **(B)** Phage maintenance was measured in the indicated strain*s* after 5 h of growth in LB without selection. Serial dilutions of 10 were dropped on plates with or without selection pressure as indicated. **(C)** Relative efficient of RS2 integration in *lac*Z::*dif*1, *lac*Z::*dif*1 ∆*hupA* and *lac*Z::*dif*1 ∆*hupB* cells. Integration was monitored after overnight growth in LB at 37°C. Data represents the mean and standard deviation of 3 independent experiments. A t-Test was used to determine the probability, *p*, that the samples came from similar distributions (*, *p*<0.1; ***, *p*<0.001). **(D)** Relative efficiency of integration of a non-replicative vector harbouring the *attP* of CTXϕ delivered by conjugation in *lac*Z::*dif*1, *lac*Z::*dif*1 ∆*hupA*, *lac*Z::*dif*1 ∆*hupB*, *lac*Z::*dif*1 ∆*hupAB* cells. Integration was monitored directly after conjugation. Data represents the mean and standard deviation of 3 independent experiments. **(E)** Relative production of CTXϕ particles by ∆*hupB* ∆*xerC* cells. ∆*xerC* recipient cells were incubated for 20’ in the filtered supernatant ∆*xerC* and ∆*hupB* ∆*xerC* donor cells harbouring pCTX-Kn. Data represents the mean and standard deviation of 3 independent experiments. The detection limit of the experiment is indicated by a dotted line.

The amplification of the phage genome by rolling-circle replication (RCR) is central to this life cycle (Fig 1): once delivered in the cytoplasm of the cell via interactions with TCP and the TolQRA cell division proteins (Fig 1, (1)) [5,9], the circular single-stranded DNA (ssDNA) genome of CTXϕ is converted into a double stranded DNA (dsDNA) replicative form by the host machinery, which permits its RCR amplification and the production of new phage particles [10,11] (Fig 1, (2), (3) and (4)). In addition to phage particle production, RCR participates in the vertical transmission of *ctxAB* in the lineage of infected cells (Fig 1). However, vertical transmission is also assured by the integration of CTXϕ into the genome of its host [5] (Fig 1). CTXϕ exploits a chromosomally encoded site-specific recombination (Xer) machinery for integration [12,13] (Fig 1). The Xer machinery normally serves to resolve dimers of the circular chromosomes by the addition of a crossover at a specific site, *dif* [14,15]. In *V*. *cholerae*, as in most bacteria, it consists of two tyrosine recombinases, XerC and XerD. The attachment site of the phage, *attP* ^*CTX*^, consists in the stem of a hairpin of its single stranded DNA genome [16,17] (Fig 1). XerC catalyses the formation of a Holliday Junction (HJ) between *attP* ^*CTX*^ and the *dif* site of one or the other of the two circular chromosomes of *V*. *cholerae* [16,17] (Fig 1 (6)). Replication converts the HJ intermediate into product [16–18]. The process is facilitated by EndoIII, a host-encoded base excision repair enzyme, which inhibits XerC catalysis once the HJ has been formed [18] (Fig 1, (7)). Nevertheless, the integration of non-replicative forms of CTXϕ is inefficient [18]. In contrast, the integration of replicative forms is very efficient and almost always leads to multiple tandem insertions, which suggests that it occurs after several rounds of amplification of the phage genome by RCR [18,19] (Fig 1). Multiple tandem insertions are permitted because a functional *dif* site is re-created on the right side of the prophage [13] (Fig 1). Tandem insertions are crucial for the life cycle of CTXϕ because the Xer recombination site on the left side of the prophage is masked in the dsDNA from of the prophage, which impedes excision [16] (Fig 1). Production of new free copies of the phage genome then depends on a process analogous to RCR between tandem prophage copies [11] (Fig 1, (8)).

**Fig 5:**
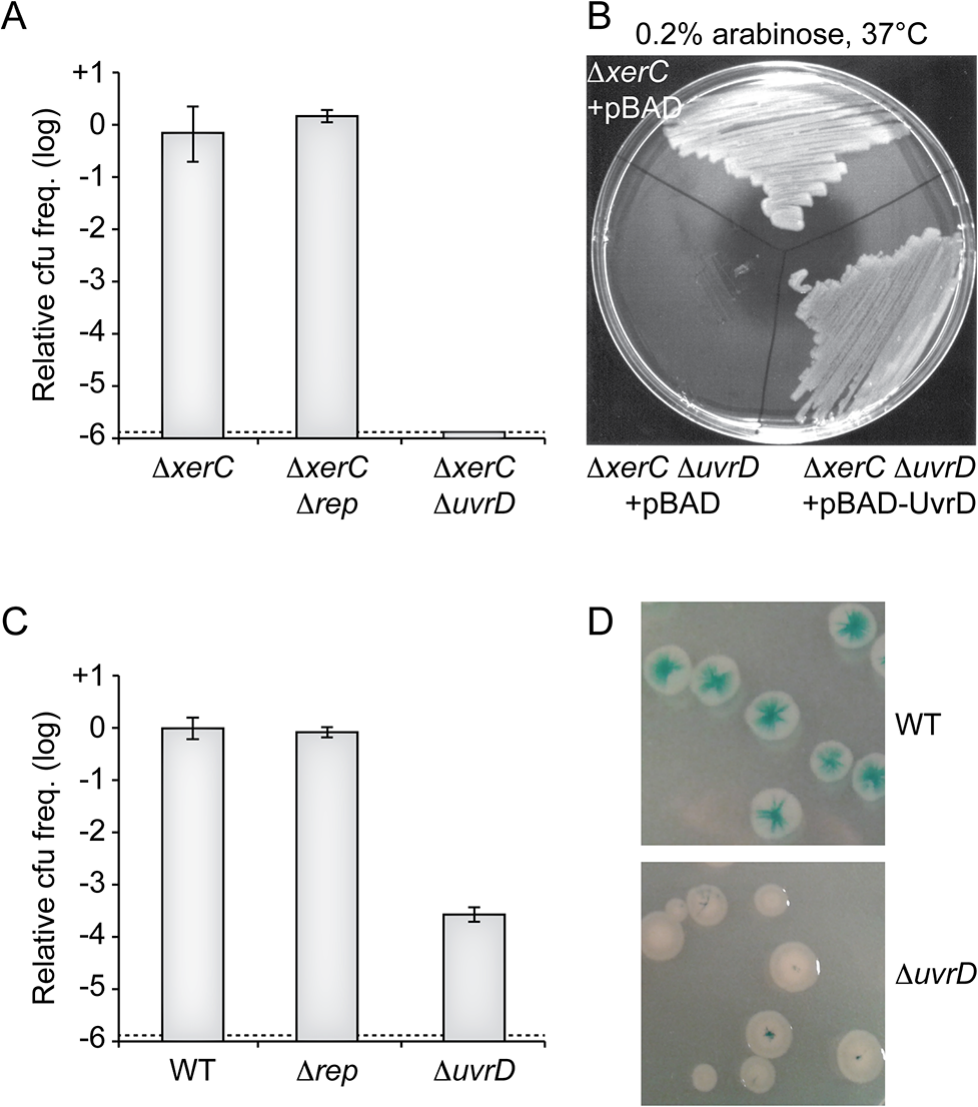
Replication of CTXϕ depends on UvrD. **(A)** Relative colony-forming ability after conjugation with RS2 in the indicated strain*s*. After 3 h of conjugation serial dilutions were plated on chloramphenicol and incubated overnight at 37°C. Relative colony forming units (cfu) correspond to the ratio of the number of colonies obtained in the indicated strain over the mean number of colonies obtained in ∆*xerC* cells. Results are shown in a logarithmic scale and represent the mean and standard deviation of 3 independent experiments. The detection limit of the experiment is indicated by a dotted line. **(B)** Complementation assay of Δ*xerC* Δ*uvrD* cells with a pBAD vector carrying the *V*. *cholerae uvrD* gene (pBAD-*uvrD*). RS2 was conjugated into Δ*xerC* and Δ*xerC* Δ*uvrD* cells harbouring pBAD or pBAD-*uvrD* and plated on LB supplemented with ampicillin, chloramphenicol and 0.2% of arabinose. Plates were incubated overnight at 37°C. **(C)** Relative colony-forming ability of *lac*Z::*dif*1, *lac*Z::*dif*1 Δ*rep* and *lac*Z::*dif*1 Δ*uvrD* cells after 3 h of conjugation with RS2. Serial dilutions were plated on chloramphenicol and incubated overnight at 37°C. Relative colony forming units (cfu) correspond to the ratio of the number of colonies obtained in the indicated strain over the mean number of colonies obtained in Δ*xerC* cells. Results are shown in a logarithmic scale and represent the mean and standard deviation of 3 independent experiments. The detection limit of the experiment is indicated by a dotted line. **(D)** Phenotype of colonies obtained after RS2 integration in *lac*Z::*dif*1 (Top) and *lac*Z::*dif*1 Δ*uvrD* (Bottom) cells.

CTXϕ RCR depends on a single phage-encoded protein, RstA (Fig 1). RstA production is under the control of the host SOS response [20] (Fig 1, (5)) and of a phage-encoded repressor, RstR [21]. RstA is an HUH endonuclease [22]. It creates a 5′-phosphotyrosine intermediate and a free 3′-OH at a specific cleavage site of the replicative form of CTXϕ, *ori(+)*, to prime replication (Fig 1). The rest of the process is driven by the host machinery [21]. Host factors implicated in the replication of the *E*. *coli* filamentous phages are either essential, such as DNA polymerase III, or crucial to the proliferation of the cells, such as the Rep helicase [10,19]. However, marked differences in the life cycles of CTXϕ and of the proto-typical filamentous phages of *enterobacteriaceae*, including its ability to integrate into the genome of its host, the control exerted by the host SOS response on RstA production [20] and the requirement for a host-encoded protein for CTXϕ particle secretion [23], suggested that it might not be so for CTXϕ. Here, we screened for non-essential host factors involved in CTXϕ replication. We thus found that the histone-like protein HU [24] was essential for CTXϕ replication because it was necessary for RstA to introduce a nick in the phage genome at *ori(+)*. We further found that in place of Rep, CTXϕ exploited UvrD, a DNA helicase mainly involved in DNA repair [25]. Finally, we found that HU and UvrD were implicated in the replication of other Vibrio filamentous phages, such a VGJϕ.

## Results

### Screening strategy

We previously described a colorimetric assay to monitor IMEX integration events in *V*. *cholerae* [17]. In brief, the *dif* site of the largest of the two chromosomes harboured by the *V*. *cholerae* N16961 El Tor strain, *dif1*, was inserted in the coding region of the *Escherichia coli lacZ* gene in such a manner as not to perturb β-galactosidase production. The *lacZ*::*dif1* allele was inserted in place of the normal *dif1* site of a N16961 El Tor strain in which the endogenous *lacZ* gene was deleted (Fig 2A). This strain forms blue colonies on X-gal media. However, 100% of the colonies obtained after the delivery of a truncated form of the El Tor variant of CTXϕ, RS2, which is fully functional in replication and integration, were white or contained large white sectors around a blue star shaped centre on X-gal plates (Fig 2B, panel *(i)* and *(ii)*). We previously used this property to search for non-essential host factors implicated in the integration of CTXϕ by transposition mutagenesis (Fig 2B, panel *(iii)*, [18]). During the course of this first screen, we noted that fully white colonies represented a very limited fraction of the total colonies, confirming the importance of ssDNA amplification by RCR for the integration process (Fig 2B, panel *(i)*). It suggested that the assay could be used in a second screen to identify non-essential host factors involved in RCR (Fig 2B, panel *(iv)*). To this end, we cloned RS2 on a pSC101 plasmid that harboured a spectinomycin resistance gene and that could be delivered by conjugation (Fig 2A). By using a temperature-sensitive version of the pSC101 origin of replication, we could distinguish if the absence of integration was due to the disruption of host factors implicated in RCR or in the integration process (Fig 2B, panel *(iii)* and *(iv)*). As a control, we verified that conjugation of the pSC101-RS2 hybrid in ∆*xerC* cells yielded fully blue colonies at 30°C and 42°C. We also verified that disruption of RstA, which abolishes RCR, led to fully blue colonies at 30°C that couldn’t grow at 42°C.

We implemented the screen in two independent mariner transposition libraries of the *lacZ*::*dif1* reporter strain. Conjugants were selected on plates supplemented with spectinomycin and X-gal at 30°C. We screened over 40 000 clones. Only 6 of them were both fully blue on X-gal plates and thermo-sensitive. All of them carried a transposon insertion in the VC1919 ORF of the V. cholerae genome (Fig 2C). Sequence analysis revealed that they corresponded to at least three independent transposition insertion events (Fig 2C).

### HU is essential for CTXϕ replication

In *E*. *coli*, HU is composed of two subunits, HUα and HUβ, which are encoded by *hupA* and *hupB*, respectively [24]. The major form of HU is a heterodimer of HUα and HUβ, but HUα homo-dimers and HUβ homo-dimers are also formed. VC1919 encodes for a homologue of the β subunit of *E*. *coli* HU, HUβ. A homologue of the α subunit of *E*. *coli* HU, HU α, is encoded by VC0273. We engineered His-tag versions of the two gene products under their native promoters and showed that they were produced at the same level at 37°C and 42°C (S1 Fig). We purified the recombinant proteins and showed that they bound DNA with similar affinities (S2 Fig). These results suggested that VC0273 and VC1919 were the *V*. *cholerae* orthologs of *E*. *coli hupA* and *hupB*.

To confirm the results of our screen, we delivered a version of RS2 marked with a chloramphenicol resistance gene in a Δ*hupB* Δ*xerC* strain by conjugation. Note that, contrary to pSC101-RS2, this version of the phage does not contain a functional plasmid origin of replication. Because of the absence of XerC, RS2 cannot integrate in this strain and vertical transmission of chloramphenicol resistance to daughter cells entirely depends on RS2 RCR. In agreement with the results of our screen, no colonies were obtained on selection plates at 42°C (Fig 3A). Colonies were obtained at 37°C (Fig 3B), but they failed to propagate when re-streaked at 42°C (Fig 3C). To further determine the potential role of HU in CTXϕ replication, we engineered a ∆*hupA* ∆*xerC* strain and a ∆*hupAB* ∆*xerC* strain. The deletion of *hupA* did not affect the maintenance of RS2 at 37°C (Fig 3B and 3D) and 42°C (Fig 3A and 3C). However, no colonies were obtained when RS2 was delivered in the ∆*hupAB* ∆*xerC* strain whether at 42°C or 37°C (Fig 3A and 3C). Ectopic production of HUα or HUβ in ∆*hupAB* ∆*xerC* cells restored colony formation at 37°C, excluding any polar effect of the two deletions (Fig 3E). Taken together, these results suggested that HU was essential for CTXϕ replication, that HUα homo-dimers were sufficient to maintain the RF of the CTXϕ genome at 37°C but that HUβ homo-dimers and/or HUαβ hetero-dimers were absolutely required at 42°C.

### The single deletion of *hupB* is sufficient to limit CTXϕ vertical and horizontal transmission

In order to gain a quantitative measure of the importance of HUα and HUβ in the CTXϕ replication process, we used quantitative PCR to monitor the number of RS2 ssDNA and dsDNA copies per genome equivalent in ∆*hupA* ∆*xerC* and ∆*hupB* ∆*xerC* cells that were grown under selection pressure at 37°C. The deletion of *hupA* had no visible effect on the relative number of RS2 copies, whether ssDNA or dsDNA (Fig 4A). In contrast, the deletion of *hupB* induced a 40% reduction in the number of RS2 copies per genome (Fig 4A). As the total number of RS2 copies per genome equivalent was now lower than 1, we suspected that the deletion of *hupB* would increase the instability of RS2 at 37°C even though it did not compromise colony formation on selection plates at this temperature. Indeed, a 100-fold reduction in the number of colony forming units was observed in ∆*hupB* ∆*xerC* cells compared to ∆*hupA* ∆*xerC* or ∆*xerC* cells after 5 hours of growth without selection pressure (Fig 4B). Because it limited the number of copies of the ssDNA CTXϕ genome, we further suspected that the deletion of *hupB* would also prevent RS2 integration. Indeed, we observed a 5-fold reduction in the integration efficiency of RS2 in ∆*hupB lacZ*::*dif1* cells compared to *lacZ*::*dif1* cells (Fig 4C). A weaker, yet significant, decrease in RS2 integration was also observed in ∆*hupA lacZ*::*dif1* cells (Fig 4C). No decrease in the frequency of integration of a non-replicative plasmid harbouring *attP* ^*CTX*^ was observed in ∆*hupA*, ∆*hupB* and ∆*hupAB*, excluding any participation of HU in the integration process *per se* (Fig 4D). Finally, we suspected that the deletion of *hupB* might also prevent the production of phage particles by limiting the amount of ssDNA available for packaging. Indeed, a 1000-fold less phage particles were produced in ∆*hupB* ∆*xerC* cells than in ∆*xerC* cells (Fig 4E). Taken together, these results suggested that the deletion of *hupB* could by itself limit CTXϕ vertical transmission via lysogenic conversion and limit horizontal transmission via the production of new viral particles.

### CTXϕ relies on UvrD for RCR

RCR of the proto-typical filamentous phages of *E*. *coli* depends on Rep, a helicase that is implicated in the replication of their host genome [26]. The *E*. *coli* Rep protein is not essential but its deletion leads to a severe growth defect [27,28]. The genome of *V*. *cholerae* encodes for a homologue of *E*. *coli* Rep. We found that it was not essential but that its deletion led to a severe growth defect, suggesting functional homology with *E*. *coli* Rep (S3 Fig). However, the deletion of *V*. *cholerae* Rep impeded neither the maintenance of RS2 in ∆*xerC* cells (Fig 5A) nor its integration (Fig 5B), suggesting that it was not implicated in CTXϕ RCR.

Some RCR plasmids of Gram+ bacteria replicate in *E*. *coli* using the UvrD DNA helicase [29]. The *E*. *coli* UvrD protein plays essential roles in methyl-directed mismatch repair and nucleotide excision repair of DNA [30]. It is also involved in clearing and restarting stalled replication forks [31–33]. It is under the control of two promoters: one is constitutive while the other is governed by LexA, which leads to a 3 to 6-fold overproduction of UvrD during SOS [34,35] (S4A Fig). *E*. *coli* UvrD is not essential and its deletion does not affect cell proliferation under normal growth conditions. The genome of *V*. *cholerae* encodes a homologue of *E*. *coli* UvrD. Its deletion did not affect cell proliferation (S4 Fig) but made them hyper sensitive to UV (S4B Fig). Inspection of the upstream region of the gene suggested the presence of two promoters, with a putative *lexA-*binding site overlapping the -10 box of one of them (S4A Fig). Correspondingly, introduction of a non-cleavable allele of *lexA* led to a 3-fold decrease in the expression of the gene (S4C Fig) while disruption of RecA or of the *lexA* box increased its expression (S4D Fig). Taken together, these results suggested that this gene was the functional homologue of *E*. *coli uvrD* and we wondered if its product was involved in CTXϕ RCR. Consistent with this view, deletion of *V*. *cholerae uvrD* almost abolished the maintenance of RS2 in ∆*xerC* cells (Fig 5A). Ectopic production of *V*. *cholerae* UvrD under an arabinose promoter on a plasmid restored colony formation, excluding any polar effect of the deletion (Fig 5B). The deletion of *V*. *cholerae uvrD* also led to over a 1000-fold drop in the frequency of integration of RS2 in XerC^+^ cells (Fig 5C). The few colonies that were obtained were fully white or only displayed a pinpoint blue dot at their centre, further indicating that integration occurred immediately after entry into the cell (Fig 5D). Taken together, these results suggested that CTXϕ relied on the UvrD helicase for RCR.

### Nicking of *ori(+)* depends on HU

**Fig 6:**
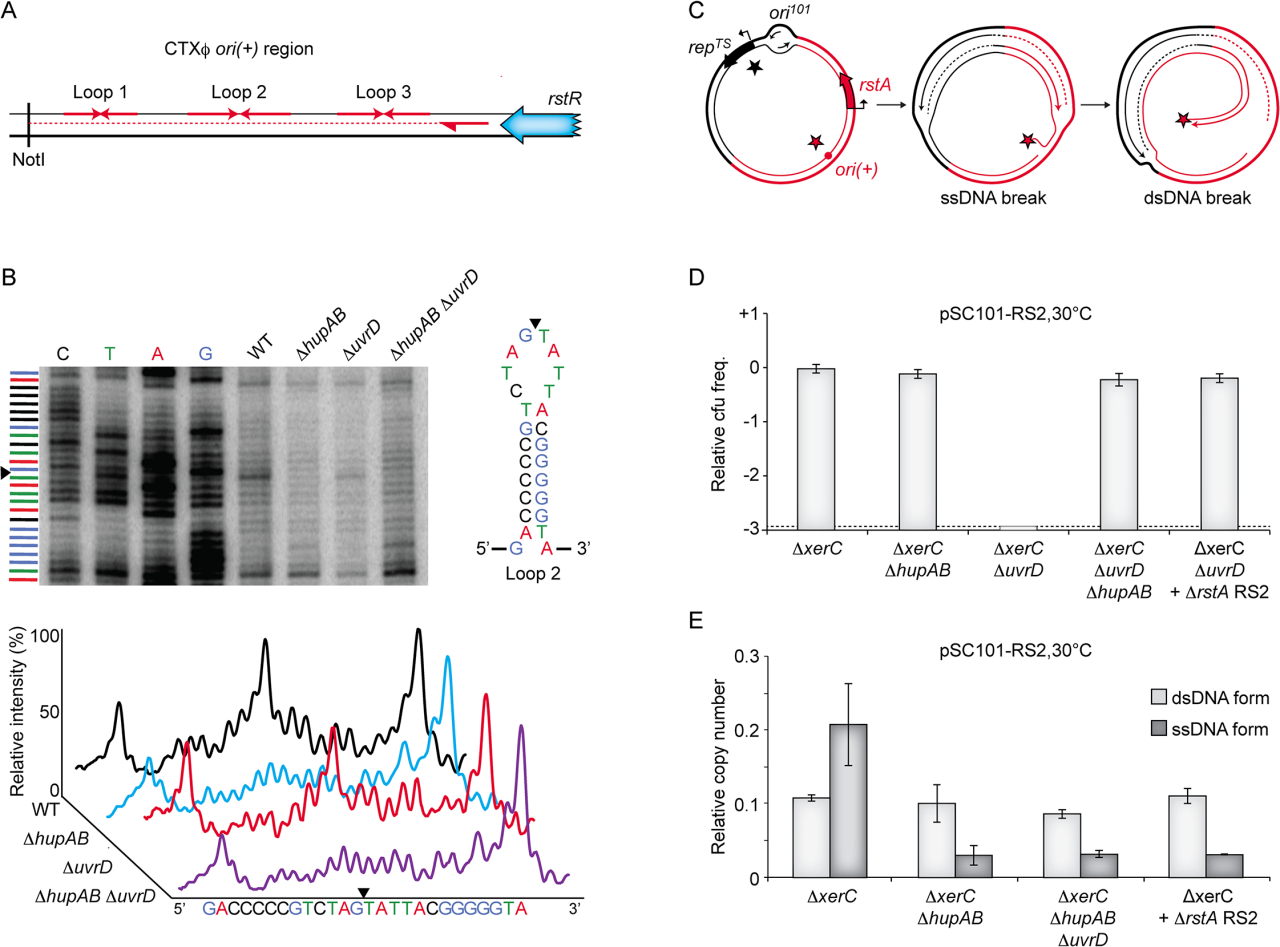
Cleavage of CTXϕ *ori(+)* by RstA depends on HU. **(A)** Scheme depicting the primer extension assay used to monitor RstA cleavage. Red arrows in opposite direction depict potential loops in the *ori(+)* region. The *rstR* gene, the NotI site and the location of the primer used in the primer extension are shown. **(B)** RstA activity in the indicated strains. Top left. Electrophoresis of the products was performed with a 6% polyacrylamide/8M urea gel. Lane 1–4: dideoxy sequence ladder. Top right: schematic representation of *ori(+)* loop 2. Bottom: relative intensity of the primer extension profiles. Black triangle: position of the nick. **(C)** Schematic representing the formation of a dsDNA break when replication forks originating from *ori* ^*101*^ encounter a nick created by RstA. Black and red stars depict Rep^*TS*^ and RstA proteins, respectively. **(D)** Relative colony-forming ability after conjugation. Column 1–3: pSC101-RS2 was conjugated in the indicated strains to Δ*xerC*, Δ*xerC* Δ*hupAB*, Δ*xerC* Δ*uvrD*, *and* Δ*xerC* Δ*hupAB* Δ*uvrD* cells, respectively; Column 5: pSC101-RS2 Δ*rstA* was conjugated in Δ*xerC* cells. After 3 h of conjugation, serial dilutions were plated on spectinomycin and incubated overnight at 30°C. Relative colony forming units (cfu) correspond to the ratio of the number of colonies obtained in the indicated strain over the mean number of colonies obtained in Δ*xerC* cells. Results are shown in a logarithmic scale and represent the mean and standard deviation of 3 independent experiments. The detection limit of the experiment is indicated by a dotted line. **(E)** Q-PCR analysis of the number of pSC101-RS2 ssDNA and dsDNA copies in Δ*xerC*, Δ*xerC* Δ*hupAB* and Δ*xerC* Δ*hupAB* Δ*uvrD* cells, and of Δ*rstA* pSC101-RS2 ssDNA and dsDNA in Δ*xerC* cells. The analysis was performed on the total DNA of cells that were grown under selective pressure at 30°C to an OD_600nm_ of 0.3. Data represent the mean of two independent experiments.

There are three different steps in RCR: (i) addition of a nick at *ori(+)* to prime replication; (ii) displacement of the old (+) ssDNA copy of the genome and synthesis of a new one; (iii) termination of replication and re-circularization of the old (+) ssDNA genome copy. HU could be involved in any of these steps. By definition, UvrD was expected to be only involved in the second step. To investigate whether HU and UvrD were involved in the first step of RCR, total genomic DNA was extracted from *V*. *cholerae* cells 3 hours after conjugation of RS2 and the presence of a nick at *ori(+)* was revealed by primer extension (Fig 6A and 6B). In wild-type cells, we observed a strong signal consistent with the introduction of a nick between the guanine and the thymine bases of the apical loop of the second hairpin of CTXϕ *ori(+)* (Fig 6B). The position of the observed nick fitted with previous genetic analysis of the cleavage position of RstA [36]. Nick formation was entirely suppressed when HU was deleted, suggesting that HU was essential for the activity of RstA (Fig 6B). In contrast, the deletion of UvrD did not affect nick formation, suggesting that UvrD was not implicated in RCR initiation.

One concern regarding our screening procedure was that we did not recover any transposition event in the *uvrD* gene even though it is not essential in *V*. *cholerae*. However, we found that pSC101-RS2 is not able to propagate in *∆uvrD ∆xerC V*. *cholerae* cells even at the permissive temperature (Fig 6D). We then hypothesized that replication forks originating from the pSC101 origin would generate fatal double strand breaks when they reached a nicked *ori*(+), which could explain why the pSC101-RS2 hybrid failed to propagate in Δ*uvrD ΔxerC* cells (Fig 6C). In agreement with this hypothesis, deletion of HU or inactivation of RstA restored the propagation of the pSC101-RS2 hybrid in Δ*uvrD ΔxerC* cells (Fig 6D). There was little or no production of RS2 ssDNA in such cells, further illustrating the importance of HU for RCR (Fig 6E).

### Role of HU and UvrD in the RCR of other *V*. *cholerae* phages

**Fig 7:**
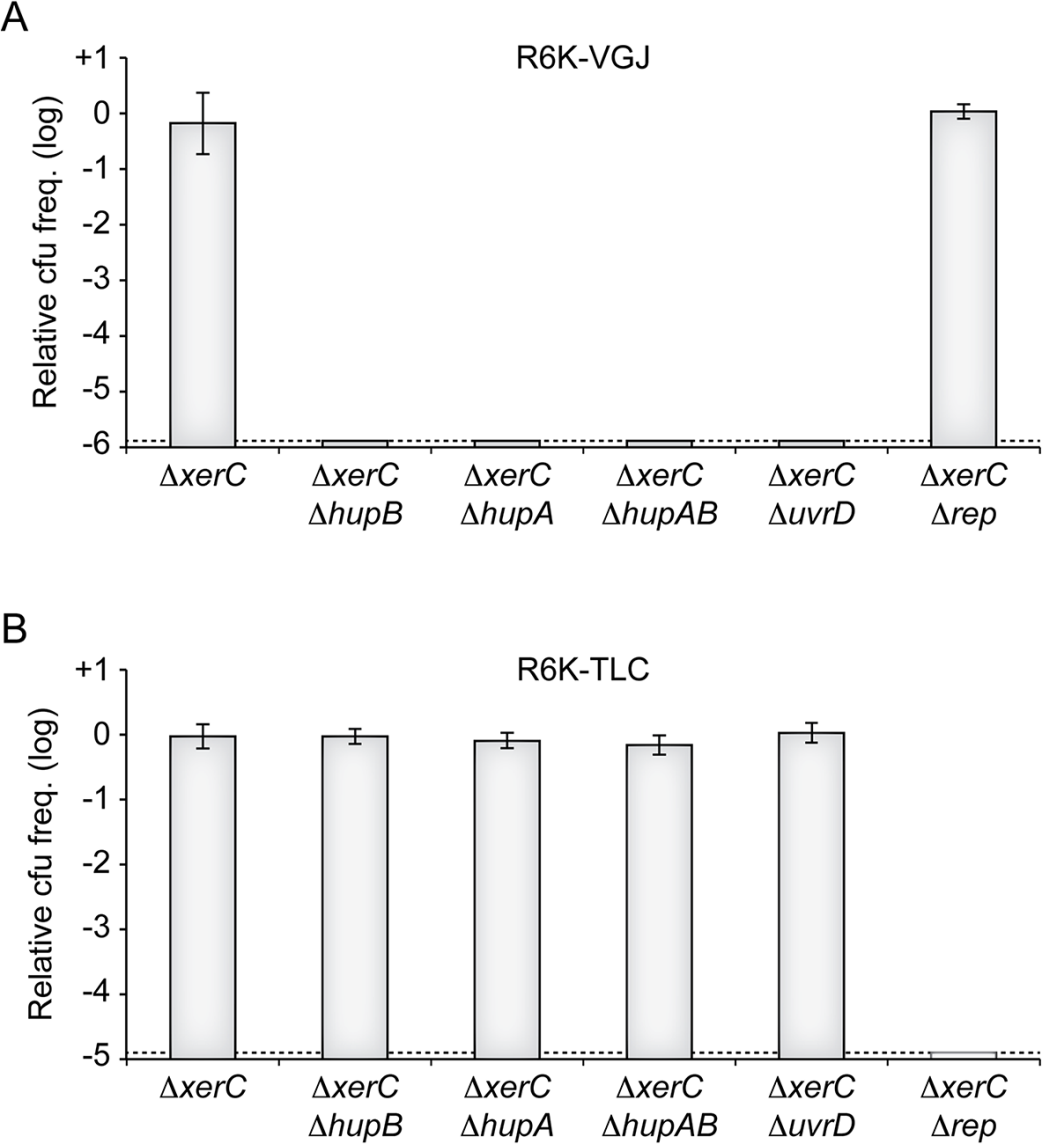
HU and UvrD in RCR of other *V*. *cholerae* IMEXs. **(A)** Relative colony-forming ability of R6K-VGJ in the indicated strains. **(B)** Relative colony-forming ability of R6K-TLC in the indicated strains. Relative colony forming units (cfu) correspond to the ratio of the number of colonies obtained in the indicated strain over the mean number of colonies obtained in ∆*xerC* cells. Results are shown in a logarithmic scale and represent the mean and standard deviation of 3 independent experiments. The detection limit of the experiment is indicated by a dotted line.

Ecological interactions between CTXϕ and several other filamentous phages and their satellites drives the continuous and rapid emergence of new epidemic variants of *V*. *cholerae* [13,15]. Foremost among the phages implicated in those interactions are RS1, which encodes for an anti-repressor [37,38], VGJϕ, which participates in the horizontal spreading of CTXϕ via the formation of CTX-VGJϕ hybrids [39,40], and TLCϕ, which is almost always found integrated before CTXϕ prophages in clinical isolates and which can lead to their excision [41–43]. We could easily predict that RS1 depended on HU and UvrD for replication, because it is essentially identical to RS2. To determine if VGJϕ and TLCJϕ might also depend on HU and UvrD, we conjugated a R6K suicide vector harbouring the replicative region of VGJϕ (R6K-VGJ) and a R6K suicide vector harbouring the replicative region of the satellite phage TLCϕ (R6K-TLC) in ∆*xerC* cells in which *hupA*, *hupB*, *uvrD* or *rep* were disrupted (Fig 7). No colonies were obtained when R6K-VGJ was conjugated in *hupA* or *hupB* mutants, suggesting that the HUαβ heterodimer was vital to VGJϕ RCR (Fig 7). R6K-VGJ also failed to be propagated in ∆*uvrD* cells, suggesting that UvrD was required for VGJϕ RCR (Fig 7). In contrast, ∆*hupAB* cells and ∆*uvrD* cells seemed to fully support TLCϕ replication (Fig 7). Finally, R6K-TLC was not maintained in ∆*rep* ∆*xerC* cells, suggesting that TLCϕ RCR depended on Rep (Fig 7).

## Discussion

We developed a screening strategy to identify non-essential *V*. *cholerae* host factors involved in CTXϕ replication. We thus found that contrary to the proto-typical filamentous phages so far studied, the histone-like HU protein was absolutely necessary for RstA to prime RCR of the phage genome (Fig 2, 3 and 6). In addition, we showed that CTXϕ exploited UvrD, a helicase normally implicated in DNA repair, rather than Rep, the helicase normally associated to replication (Fig 5). Finally, we showed that a member of another family of vibrio filamentous phages, VGJϕ, also exploited HU and UvrD for RCR, demonstrating that CTXϕ is not an exception (Fig 7).

### A role for HU in RCR

HU is a major component of the bacterial nucleoid, which binds dsDNA without any apparent specificity and with a low affinity but which recognizes with a higher affinity defined DNA structures and repair intermediates [44–46]. In *E*. *coli*, HU is involved in the initiation of chromosome replication [47–49]. However, it is not essential for survival: IHF, a protein belonging to the same family of DNA-binding proteins, can substitute for initiation of replication at *oriC* [50]. Likewise, deletion of *hupAB* does not compromise cell viability in *V*. *cholerae*, possibly because its genome encode for a homologue of IHF.

As far as we know, no reports exist on the implication of HU in the life circle of any other filamentous phages than CTXϕ and VGJϕ. HU was shown to be essential for replication of Mini-F and Mini-P plasmids [51]. However, these plasmids replicate by a theta system. In this case, HU bind to the origin without sequence-specificity and help to melt the origin to initiate replication [52]. Interestingly, it was observed in *Salmonella typhimurium* that replication of a Mini-F plasmid was strongly affected in a ∆*hupB* mutant, totally deficient in a ∆*hupAB* double mutant, but only mildly affected in a ∆*hupA* mutant [53]. This is remarkably similar to what we have observed in the case of CTXϕ and a similar role of HU in the initiation of replication should not be discarded. More interestingly, however, it was reported that HU played an essential role in the replication of pKYM, a plasmid from the Gram^-^ bacterium *Shigella sonei* [54]. A shared characteristic of proto-typical filamentous phages and of most RCR plasmids is a very simple (+) origin of replication: Ff coliphages contain an approximately 36bp replication origin [55]; the Gram^+^ pC194 and pT181 plasmids harbour a small 55bp and 70bp origin, respectively [56,57]. None of these mobile elements require accessory proteins for the initiator protein nicking activity. In contrast, pKYM and CTXϕ (+) origins of replication are more complex. The (+) origin of replication of pKYM is 173bp long. It contains a core region corresponding to the RepK initiator binding-site and a downstream enhancer region. HU was shown to specifically recognize this enhancer region and assist in the binding of RepK [54]. CTXϕ *ori(+)* is 167bp long and contains several inverted repeat sequences upstream and downstream of the RstA cleavage site with the potential to form stem-loops [36]. It is therefore possible that HU helps CTXϕ replication by helping the binding of RstA and/or promoting its endonuclease activity. A weaker binding affinity and/or tighter control of the VGJϕ HUH endonuclease might explain why the two HU subunits are absolutely essential for this phage. Future biochemical work will need to clarify the exact mechanism of action of HU on RstA activity.

### Implication of UvrD in RCR

Rep and UvrD are members of the SF1 family of helicases and share approximately 40% similarity [58]. They both unwind DNA in the 3’ – 5’ direction [59,60]. Despite the structural and functional similarities between Rep and UvrD, the physiological roles of the two helicases are well distinct. Rep is constitutively expressed in *E*. *coli*, where it is implicated in chromosome replication: it directly interacts with the replicative helicase DnaB and helps remove nucleoproteins complex in front of replication forks [61,62]. Rep is also implicated in the restart of stalled replication forks [63]. As a result, Δ*rep E*. *coli* mutants display a 50–60% reduction in their replication rate [27,28]. Nevertheless, Rep is not essential. On the contrary, UvrD is overexpressed during the SOS response in *E*. *coli* and its role seems to be mainly limited to DNA repair: its activity is involved in MutHLS-dependent mismatch DNA repair [64] and UvrABC-dependent nucleotide excision repair [65]. UvrD also helps dismantle RecA filaments from ssDNA, which prevents unwanted recombination [66]. Finally, UvrD can promote the movement of the replisome along protein-bound DNA and participate in the restart of replication forks [62]. Nevertheless, its deletion does not directly affect replication fork progression in *E*. *coli* [61]. Consistent with its role in replication fork progression, Rep was shown to be critical for phage RCR in *E*. *coli*, including ϕX174 and the Ff family of filamentous phages [26]. In contrast, we found that CTXϕ and VGJϕ both exploited UvrD for RCR. As far as we know, this is the first time that UvrD has been shown to participate in the replication of a phage genome. A single SF1 helicase, PcrA, is encoded in the genome of Gram+ bacteria instead of Rep and UvrD. RCR of plasmids from Gram+ bacteria relies on PcrA. However, some of them can replicate in *E*. *coli* using UvrD [29]. In addition, UvrD was shown to be implicated in the RCR of pKYM [67]. Together, these results suggest that RCR depends on an activity common to Rep and UvrD, raising the question as to why these two helicases are not interchangeable, similarly to PcrA and UvrD. It is tempting to speculate that exploitation of UvrD or Rep is determined by the ability of the initiator protein to directly interact with one or the other of the two accessories helicases. In agreement with this hypothesis, the initiator protein of CTXϕ and VGJϕ share structural similarities with the initiator protein of the Gram+ plasmids that exploit UvrD to replicate in *E*. *coli* (pfam02486). In contrast, the initiator protein of TLCϕ shares sequence and structural similarities with the initiator protein of the *E*. *coli* proto-typical filamentous phages (pfam05144 and pfam05155). Future work will be directed at investigating the exact nature of the interaction between UvrD and RstA.

### Considerations for the biosafety of live-attenuated vaccine cells

In 2011, recognizing that cholera was not sufficiently addressed despite its prevalence in epidemic forms in both endemic and non endemic areas, the World Health Assembly called for a comprehensive approach to cholera control, including the development of oral cholera vaccines (http://www.who.int/wer). The most promising live attenuated *V*. *cholerae* vaccine strains have been obtained by the deletion of one or both of the cholera toxin genes, *ctxAB* [68–71]. However, the possibility that such strains could be re-infected when in the intestinal track raised safety concerns about their use in a vaccine since they could promote the apparition of cholera symptoms in previously asymptomatic individuals and participate in the spreading of CTXϕ in the environment (S5A Fig). The concomitant deletion of the *dif* site of Chr1 in these strains only partially prevents *ctxAB* reacquisition since some phage variant can target the *dif* site of Chr2 [17] and does not block RCR amplification of the phage genome.

Several possibilities exist to limit the risk of re-acquisition of the genes and their further spreading. A simple way to block the delivery of the genome of CTXϕ could be to delete the production of its receptors at the cell surface, TCP and TolQRA. However, TCP is essential for intestinal colonization and hence immunogenicity [4]. TolQRA is part of the cell division machinery and is critical for the outer membrane stability of Gram^-^ bacteria and their resistance to extra-cytoplasmic stress [72–76]. A simple way to limit further spreading of CTXϕ particles could be to block their secretion by deleting EspD [23]. However, EspD appears to be essential in *V*. *cholerae* [23]. As a result, the only valid vaccine cell protection strategy proposed to date was based on the observation that production of RstR from a resident CTXϕ prophage provided immunity against secondary infections by blocking initial rounds of RCR [77] (S5B Fig). However, this strategy has several limitations. First, several CTXϕ variants exist that harbour different RstR repressors cross-immunity is not assured among them [78] (S5B Fig). Thus, this strategy is limited to known CTXϕ repressor variants, with each repressor providing immunity against secondary infections by phages encoding the same repressor (S5C Fig). Second, CTXϕ interacts with other Integrative Mobile Element exploiting Xer (IMEX). Two of them, the RS1 satellite phage and fs2, harbour an anti-repressor, RstC [37,79] (Fig 7B). Third, hybrid phage formation between CTXϕ and other IMEXs, such a VGJϕ, can circumvent both the requirement for TCP expression and repressor immunity [39,40,80,81] (Fig 7B). Fourth, tandem CTXϕ genomes can be transduced by lytic phages, such as CP-T1 [82]. Finally, production of RstR does not affect the efficiency of the RCR process once it has been established, which permits production of new phage particles and further spreading of CTXϕ (S5D Fig).

Here, we showed that the deletion of *hupB* impedes *ctxAB* re-acquisition by CTX-VGJϕ hybrid infection and dramatically reduces CTXϕ production when its genome has been acquired by other horizontal transfer mechanisms (S5C Fig). Therefore, we think that the deletion of *hupB* would considerably increase the safety of RstR-producing vaccine cells. Moreover, we found that HU and UvrD were both essential for CTXϕ and VGJϕ replication, that their deletion compromised the ability of CTXϕ to integrate into the genome of its host and blocked the secretion of CTXϕ particles. HU is not essential for the proliferation of *V*. *cholerae* but we cannot discard a possible impaired colonization of the HU null mutants. However, *Salmonella enterica* strains lacking *hupA* and/or *hupB* are known to trigger an effective immune response protecting against salmonellosis, suggesting that HU is probably not essential for intestine colonization [83]. Therefore, the deletion of *hupA* and *hupB* is a promising strategy for the development of safe live attenuated cholera vaccines. UvrD participates in DNA mismatch repair, many genes of which have been shown to be important for colon colonization [84]. However, in the case the deletion of *uvrD* affects colon colonization, mutating it in such a way as to compromise its role in RCR without affecting its DNA repair activities could offer a third strategy for the development of safe live attenuated cholera vaccines.

## Materials and Methods

### Strains, plasmids and oligonucleotides

Strains, plasmids and oligonucleotides used in this study are described in S1, S2 and S3 Tables, respectively. All *V*. *cholerae* strains were constructed by natural transformation. Engineered strains were confirmed by PCR and sequencing. Bacterial strains were grown on Luria-Bertani (LB) agar. Antibiotics were used at the following concentrations: ampicillin (Amp), 100 μg/mL; spectinomycin (Sp), 100 μg/mL; chloramphenicol (Cm), 34 μg/mL for *E*. *coli* and 3 μg/mL for *V*. *cholerae*; kanamycin (Kn), 50 μg/mL; Zeocin (Zeo), 100 μg/mL for *E*. *coli* and 1 μg/mL for *V*. *cholerae* and rifampicin (Rif), 100 μg/mL for *E*. *coli* and 2 μg/mL for *V*. *cholerae*. 0.2% arabinose was used to induce UvrD production from the pBAD24 vector.

### Mariner transposon-mutagenesis, screening and mutant characterization

A mariner transposon-mutagenesis bank of a *V*. *cholerae* reporter strain was created as described [18]. The bank was conjugated with a spectinomycin resistant (SpecR) derivative of RS2 El Tor containing a thermosensitive (TS) origin of replication (pSC101-RS2). Individual colonies were selected on X-Gal, IPTG and spectinomycin plates after 48 h of growth at 30°C. Fully blue colonies were selected and re-streaked in parallel at 30°C and 42°C. TS clones were cured from pSC101-RS2 by overnight growth in the absence of antibiotic and their phenotype was corroborated by re-conjugation with the same plasmid. The insertion was mapped by direct sequencing of the DNA flanking the point of insertion of the mariner transposons, which was amplified by arbitrary-random PCR [85].

### Conjugation assay

*E*. *coli* β2163 meso-diaminopimelic acid (DAP) auxotroph donors and *V*. *cholerae* recipients were grown to 0.3 at OD_600nm_. Bacteria were pelleted by centrifugation, re-suspended in 50 μL and mixed at a 1:10 ratio, dropped onto sterile filter paper on top of an LB-agar plate supplemented with DAP and incubated for 3 h. Conjugants were selected for the plasmid antibiotic resistance and DAP prototrophy. To monitor integration, conjugants were spread on plates containing X-gal and incubated at 37°C overnight. Conjugants carrying a TS origin of replication were re-covered at 30°C.

### Assay of CTXϕ infection efficiency and phage production

Strains harbouring kanamycin-marked CTXϕ were used as donors. Eighty microliters of filtered supernatant containing CTX-Kn particles was mixed with 20 μl of recipients strains that had been grown in AKI media to induce TCP expression [86]. The mix was incubated 20 min at 37°C to allow infection and then plated on LB to determine the number of potential recipients and LB supplemented with kanamycin to determine the number of infected cells. The frequency of infection was determined by the ration of Kn^R^ cells and the total number of recipients.

### Q-PCR analysis

Total DNA was purified using the GenElute Bacterial Genomic DNA Kit from Sigma. Samples were analysed using a LightCycler FastStart DNA masterSYBR Green I system from Roche. Reactions were run in triplicate using a LightCycler 480 instrument (Roche). Primer 2690 and 2704, which amplify a specific 150 bp fragment inside *rstA* gene, were used for phage DNA quantification. Data were normalized with the bacterial chromosome using primers 768 and 769, which amplify a 150 bp fragment within the *matP* gene. For single strand DNA quantification, total DNA was digested 3 hours with ScaI to remove phage dsDNA. There is a cleavage site for ScaI within the phage fragment used for the analysis. Relative copy number of ssDNA was calculated as follows: 2 x e_1_^Cp_digested^/e_2_^Cp_chromosome^, in which e represents the amplification efficiency of the primers pairs used. A factor of 2 was used to normalize the ssDNA of the phage with the dsDNA of the chromosome. The analysis was run out in parallel without prior digestion, which permitted to calculate the relative copy number of dsDNA as follows: (e_1_^Cp_undigested^-e_1_^Cp_digested^)/e_2_^Cp_chromosome^.

### SDS-page and western blot

Bacterial lysates were electrophoresed on 12% SDS-page gel. HUα or HUβ with a C-terminal 6xHis tag were analysed by western blot with a primary anti-4His mouse monoclonal antibody (Invitrogen) and a secondary anti-mouse IgG antibody coupled to peroxidase (Pierce). ECL Western Blotting Substrate (Pierce) was used to detect the reaction on a LAS-3000 Luminescent Analyser (Fujifilm).

### Sequencing gel and nick detection

For nick detection, pBS66 was conjugated to the strain of interest and then total DNA was purified directly from the conjugation assay. After digestion with NotI, we performed a primer extension reaction using as a primer the 1269 oligonucleotide that had been labelled with γ-[32P] ATP. The sequence ladder was prepared using pBS66 purified from *E*. *coli*, in which CTXϕ does not replicate, and the fmol DNA Cycle Sequencing System (Promega).

## Supporting Information

S1 Fig Equal production of HUα and HUβ at 37°C and 42°C. Western blot analysis of His-tagged HUα and HUβ. *V*. *cholerae* containing a His-tagged version of HUα or HUβ was growth to an OD_600nm_ of 0.5. The cell lysates were loaded onto an SDS-PAGE gel. Proteins were transferred to a PVDF membrane and blocked with 5% milk in TBST for 1 hour. The membrane was probed with a 4x-His antibody.
(PDF)

S2 Fig HUα and HUβ bind DNA with equivalent affinity. *In vitro* HUα and HUβ binding assay on RS2 DNA. Black triangles depict increasing concentration of HU (1.5 ng, 7.5 ng, 15 ng, 75 ng, 150 ng, 750 ng, 1500 ng) in each lane.
(PDF)

S3 Fig Deletion of the *rep* gene leads to a severe growth defect in *V*. *cholerae*. Growth curve of wild type, Δ*uvrD* and Δ*rep V*. *cholerae* cells. The strains were grown in rich media LB at 37°C and the OD_600nm_ of each culture were measured over the course of time.
(TIF)

S4 Fig *V cholerae uvrD* is under the control of the SOS response. **(A)** Top: Scheme of the *E*. *coli* and *V*. *cholerae uvrD* promoter regions. The angled arrows depict the *uvrD* transcription start sites. Red boxes show LexA binding sites. Green boxes show predicted -35 and -10 core promoter elements. Bottom: Comparison of the sequence of *E*. *coli* and *V*. *cholerae* putative LexA binding sites in *uvrD* promoter. The mutated LexA binding site of *V*. *cholerae* is shown. **(B)** UV sensitivity of Δ*uvrD* cells. Cells were grown overnight on plates and then re-suspended in minimal media M9 for UV irradiation. Top: Control without UV exposition. Bottom: Cells irradiated up to UV doses of 25 J/m^2^. **(C)** β-gal activity (Miller units) of strains harbouring a *lac*-gene transcriptional fusion to wild type or mutated *uvrD* promoters. Top: MV57 and MV57 Δ*recA*. Bottom; MV47 and MV47 *lexA* ^*ind*^ **. **
(TIF)

S5 Fig *hupB* deletion limits CTXϕ horizontal transmission. **(A)** Scheme of the re-infection of a vaccine strain by CTXϕ in the intestinal track. The vaccine strain is depicted in blue and the pathogenic strain in red. Red points depict CTXϕ particles. **(B)** Scheme of the mechanism of action of phage immunity and its limitations for the protection of cholera vaccine strains. RstR production from a resident cholera vaccine genome represses RstA production and therefore provides immunity against secondary infections by a phage harbouring the same immunity region (CTX1). RS1 satellite phage-encoded RstC anti-repressor counteracts the activity of the resident RstR. Classical CTXϕ (CTX^cl^) and other variants of CTXϕ (CTX2) contain a heterologous immunity regions which is not recognized by El Tor RstR. Hybrid CTX-VGJ phages escape RstR immunity by using the VGJϕ RCR module. **(C)** Relative susceptibility to CTXϕ infection. Donor: Δ*xerC* + CTX-Kn; Recipients: Δ*xerC*, Δ*xerC dif1*::El Tor RS2, Δ*xerC dif1*::Classical RS2 and Δ*xerC* Δ*hupB*. **(D)** Relative ability of CTXϕ production. Donors: Δ*xerC* + CTX-Kn, Δ*xerC dif1*::El Tor RS2 + CTX-Kn, Δ*xerC dif1*::Classical RS2 + CTX-Kn and Δ*xerC* Δ*hupB* + CTX-Kn. Donor strain was growth on LB media 5 hours. Filtered supernatant containing CTX-Kn particles was mixed the recipients strains which was growth in AKI media. After infection the strains were plated on LB supplemented with Kn. The number of CFU are shown in a logarithmic scale and represent the mean and standard deviation of 3 independent experiments.
(TIF)

S1 Table Strains used in the study. (DOCX)

S2 Table Plasmids used in the study. (DOCX)

S3 Table Oligonucleotides used in the study. (DOCX)
