# Supplementary figures and images for: CTXφ Replication Depends on the Histone-Like HU Protein and the UvrD Helicase
Source: PLoS Genet. 2015 May 20;11(5):e1005256. doi: 10.1371/journal.pgen.1005256 (PMC4439123; doi:10.1371/journal.pgen.1005256)

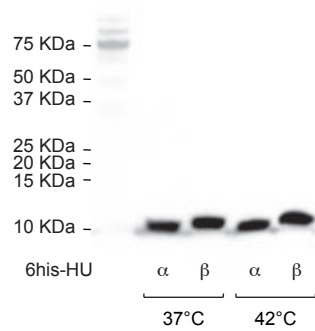

Supplement: S1 Fig — Western blot analysis of His-tagged HUα and HUβ. V. cholerae containing a His-tagged version of HUα or HUβ was growth to an OD600nm of 0.5. The cell lysates were loaded onto an SDS-PAGE gel. Proteins were transferred to a PVDF membrane and blocked with 5% milk in TBST for 1 hour. The membrane was probed with a 4x-His antibody. (PDF) [file pgen.1005256.s001.pdf]

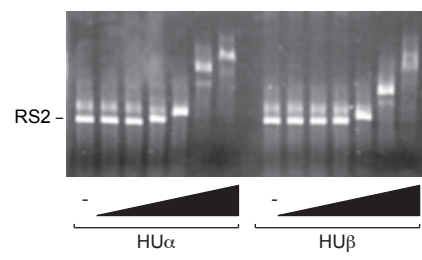

Supplement: S2 Fig — In vitro HUα and HUβ binding assay on RS2 DNA. Black triangles depict increasing concentration of HU (1.5 ng, 7.5 ng, 15 ng, 75 ng, 150 ng, 750 ng, 1500 ng) in each lane. (PDF) [file pgen.1005256.s002.pdf]

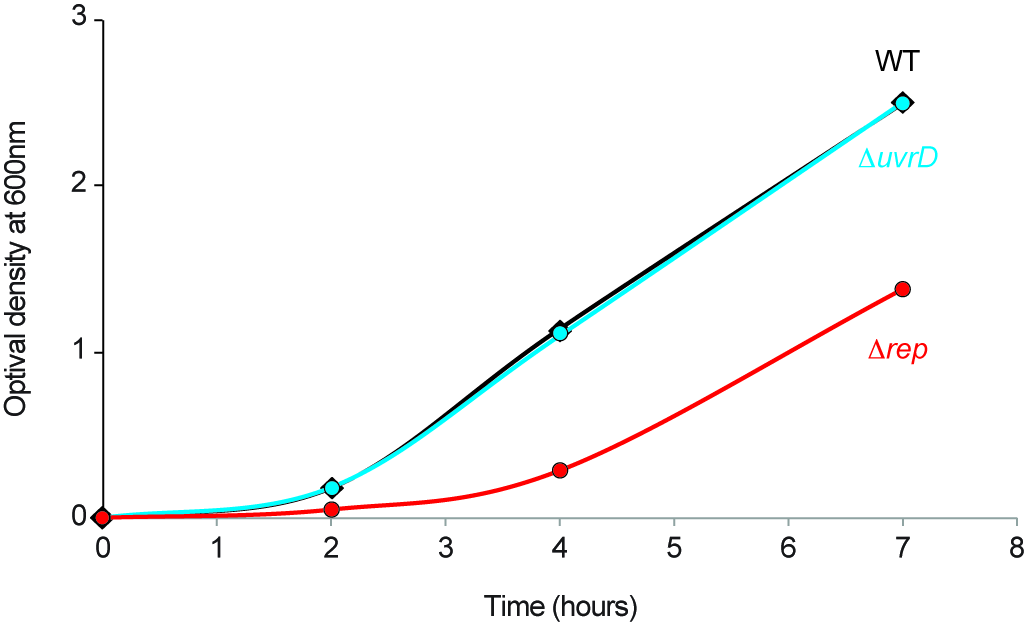

Supplement: S3 Fig — Growth curve of wild type, ΔuvrD and Δrep V. cholerae cells. The strains were grown in rich media LB at 37°C and the OD600nm of each culture were measured over the course of time. (TIF) [file pgen.1005256.s003.tif]

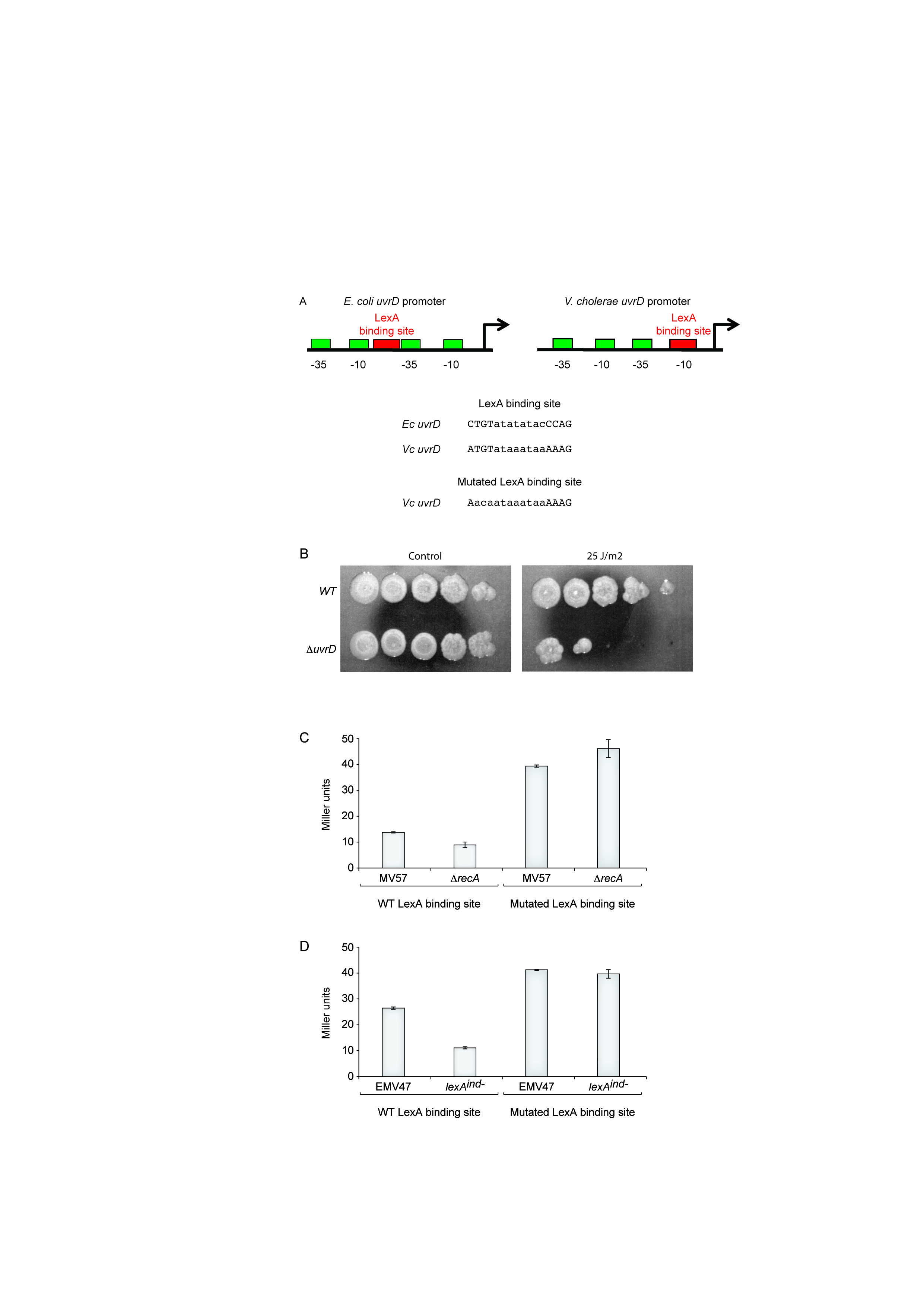

Supplement: S4 Fig — (A) Top: Scheme of the E. coli and V. cholerae uvrD promoter regions. The angled arrows depict the uvrD transcription start sites. Red boxes show LexA binding sites. Green boxes show predicted -35 and -10 core promoter elements. Bottom: Comparison of the sequence of E. coli and V. cholerae putative LexA binding sites in uvrD promoter. The mutated LexA binding site of V. cholerae is shown. (B) UV sensitivity of ΔuvrD cells. Cells were grown overnight on plates and then re-suspended in minimal media M9 for UV irradiation. Top: Control without UV exposition. Bottom: Cells irradiated up to UV doses of 25 J/m2. (C) β-gal activity (Miller units) of strains harbouring a lac-gene transcriptional fusion to wild type or mutated uvrD promoters. Top: MV57 and MV57 ΔrecA. Bottom; MV47 and MV47 lexA ind . (TIF) [file pgen.1005256.s004.tif]

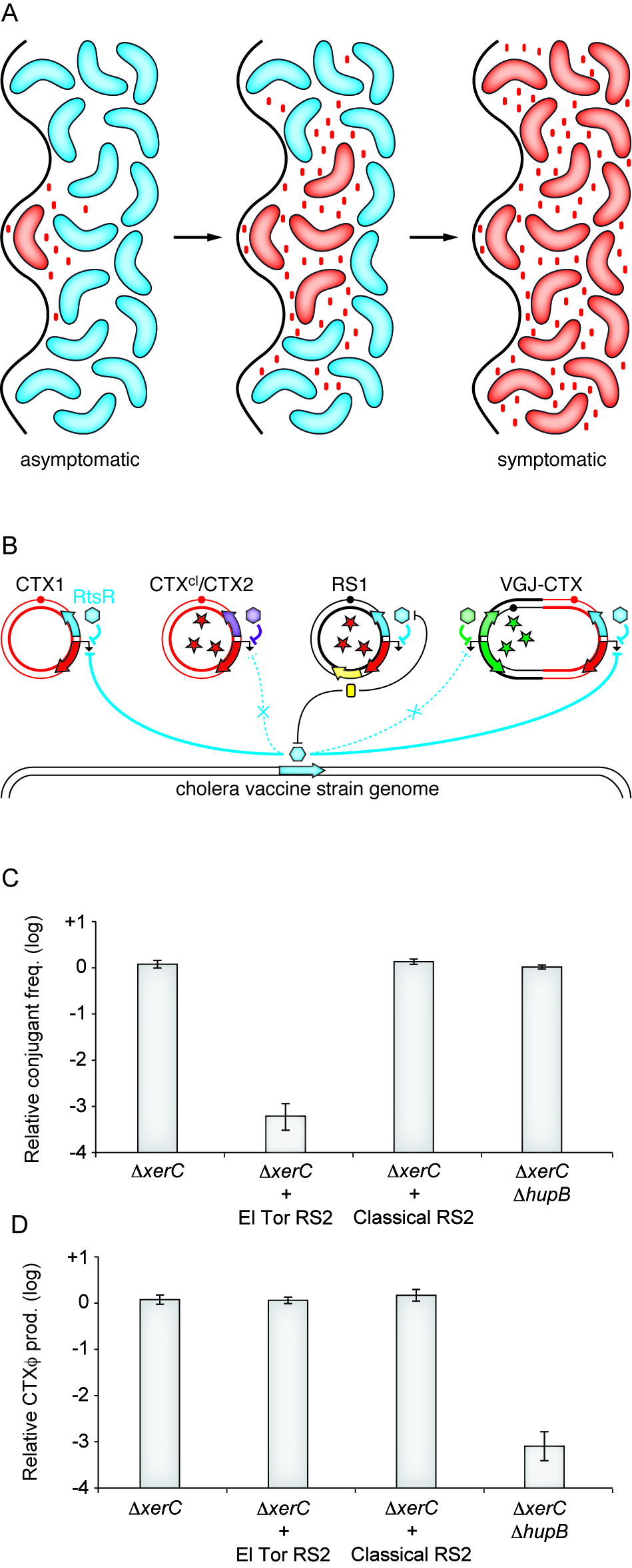

Supplement: S5 Fig — (A) Scheme of the re-infection of a vaccine strain by CTXϕ in the intestinal track. The vaccine strain is depicted in blue and the pathogenic strain in red. Red points depict CTXϕ particles. (B) Scheme of the mechanism of action of phage immunity and its limitations for the protection of cholera vaccine strains. RstR production from a resident cholera vaccine genome represses RstA production and therefore provides immunity against secondary infections by a phage harbouring the same immunity region (CTX1). RS1 satellite phage-encoded RstC anti-repressor counteracts the activity of the resident RstR. Classical CTXϕ (CTXcl) and other variants of CTXϕ (CTX2) contain a heterologous immunity regions which is not recognized by El Tor RstR. Hybrid CTX-VGJ phages escape RstR immunity by using the VGJϕ RCR module. (C) Relative susceptibility to CTXϕ infection. Donor: ΔxerC + CTX-Kn; Recipients: ΔxerC, ΔxerC dif1::El Tor RS2, ΔxerC dif1::Classical RS2 and ΔxerC ΔhupB. (D) Relative ability of CTXϕ production. Donors: ΔxerC + CTX-Kn, ΔxerC dif1::El Tor RS2 + CTX-Kn, ΔxerC dif1::Classical RS2 + CTX-Kn and ΔxerC ΔhupB + CTX-Kn. Donor strain was growth on LB media 5 hours. Filtered supernatant containing CTX-Kn particles was mixed the recipients strains which was growth in AKI media. After infection the strains were plated on LB supplemented with Kn. The number of CFU are shown in a logarithmic scale and represent the mean and standard deviation of 3 independent experiments. (TIF) [file pgen.1005256.s005.tif]
