# Supplementary material for: CTXφ Replication Depends on the Histone-Like HU Protein and the UvrD Helicase
Source: PLoS Genet. 2015 May 20;11(5):e1005256. doi: 10.1371/journal.pgen.1005256 (PMC4439123; doi:10.1371/journal.pgen.1005256)
Supplement: S1 Table — (DOCX) [file pgen.1005256.s006.docx]

**Table S1.** Strains used in this study

| Strains | Genotipe/phenotipes | References |
| --- | --- | --- |
| N16061  EMV01  EMV02  EMV03  EMV04  EMV05  EMV06  EMV07  EMV08  EMV18  EMV22  EMV37  EMV38  EMV68  EMV69 | *V. cholerae* O1 El Tor strain, St^r^  N16061 *lacZ^Ec^*::*dif1, hapA*  EMV01 *hupA::Km^r^*  EMV01 *hupB::Zeo^r^*  EMV01 *hupA::Km^r^ hupB::Zeo^r^*  EMV01 *xerC::rif^r^*  EMV02 *xerC::rif^r^*  EMV03 *xerC::rif^r^*  EMV04 *xerC::rif^r^*  EMV01 *uvrD::zeo^r^*  EMV01 *rep::zeo^r^*  EMV18 *xerC::rif^r^*  EMV22 *xerC::rif^r^*  EMV01 *dif1::pBS66 xerC::rif^r^*  EMV01 *dif1::pBS22 xerC::rif^r^* | Heidelberg *et al*., Nature, 2000  This study  This study  This study  This study  This study  This study  This study  This study  This study  This study  This study  This study  This study  This study |
