# Supplementary material for: CTXφ Replication Depends on the Histone-Like HU Protein and the UvrD Helicase
Source: PLoS Genet. 2015 May 20;11(5):e1005256. doi: 10.1371/journal.pgen.1005256 (PMC4439123; doi:10.1371/journal.pgen.1005256)
Supplement: S2 Table — (DOCX) [file pgen.1005256.s007.docx]

|  | Description | References |
| --- | --- | --- |
| pSW23T  pBS22  pBS66  pBS73  pBS90  pSW-Short-CT  pMEV245  pFX524  pEM011  pEM017  pEM019  pEM020  pEM021  pEM022  pEM029  pEM037  pEM039  pEM044  pEM062 | pSW23::oriTRP4; oriVR6Kγ; Cm^r^  pSW23T harboring the replication and integration machinery RS2 of the CTX classical phage of *V. cholerae* 569B strain; Cm^r^  pSW23T harboring the replication and integration machinery RS2 of the CTX El Tor phage of *V. cholerae* N16061 strain; Cm^r^  pSW23T harboring the replication and integration machinery of VGJ phage; Cm^r^  pSW23T harboring the replication and integration machinery of TLC satellite phage; Cm^r^  AttP from CTX El Tor phage cloned into pSW23T  pDS132 carrying an *arr*2 cassette flanked by the upstream and downstream region of *V. cholerae* xerC; Cm^r^ ,Rif^r^  pSC101 with a repAts; Amp^r^  pFX524 harboring the replication and integration machinery RS2 of the CTX El Tor phage of *V. cholerae* N16061 strain; Cm^r^, Spec^r^  pEM011 digested EcoRV, PmlI and circularized to delete RstA  pUC18 harboring hupB gene flanked by the upstream and downstream regions  pUC18 harboring hupA gene flanked by the upstream and downstream regions  pUC18 carrying an Zeo^r^ cassette flanked by  the upstream and downstream regions of  hupB; Ap^r^  pUC18 carrying an Km^r^ cassette flanked by  the upstream and downstream regions of  hupA; Ap^r^  pUC18 harboring *uvrD* gene flanked by the upstream and downstream regions  pUC18 carrying an Zeo^r^ cassette flanked by  the upstream and downstream regions of  uvrD; Ap^r^  pUC18 harboring *rep* gene flanked by the upstream and downstream regions  pUC18 carrying an *zeo^r^* cassette flanked by  the upstream and downstream regions of  *uvrD*; Ap^r^  pBAD24 harboring *uvrD* gene cloned in front of arabinose inducible promotor | Demarre *et al*. Res Microbiol. 2005  Das *et al*., PNAS, 2010  Das *et al*., PNAS, 2011  This study  Midonet *et al*., PNAS, 2014  Val et al., Mol Cell, 2005  Das *et al*., PNAS, 2010  This study  This study  This study  This study  This study  This study  This study  This study  This study  This study  This study  This study |

**Table S2.** Plasmids used in this study
