# Supplementary material for: CTXφ Replication Depends on the Histone-Like HU Protein and the UvrD Helicase
Source: PLoS Genet. 2015 May 20;11(5):e1005256. doi: 10.1371/journal.pgen.1005256 (PMC4439123; doi:10.1371/journal.pgen.1005256)
Supplement: S3 Table — (DOCX) [file pgen.1005256.s008.docx]

**Table S3.** Oligonucleotides used in this study

|  | Sequence |
| --- | --- |
| 768  769  1269  2247  2248  2251  2252  2249  2250  2253  2254  2425  2426  2427  2441  2442  2443  2690  2444  2701  2702  2704  ARB1  ARB2  ARB6  1314  846 | TAAAGTGGCGCTTACGCTTGG  GAAGCTGCGGTACAGAAGCTC  GACATTCTACCAAGAGCATC  TTGCGCTTTCAGTCCATCAG  GTGGTCTGGAGCGCGAAATC  CACGGATCCCGTCATAACTTGCGTTACTG  CACGCATGCACTCGGCAATACCGTATTAG  CACCTCGAGTCACTGTGATTCCCCTTTGG  CACAGATCTCTCTGAAAGACGCTTGCAAC  CACTCTAGATCATTTAGGTTTCCCTTCTC  CACGTCGACAGACGCGATCAAGTAATTGC  TCACGGTACCTGCGTGGCAGCTTCTATCTC  TCACTCTAGATTGGCTGCTTACCACGTCTG  TCACGGATCCTTGAGACCGTCGAGCAATAG  TCACGAATTCTCAACCGGATTGGCGGTCAC  TCACTCTAGAAACCCACGACAACCGGAATC  TCACAGATCTCCGCTTCATCTTGTCTTGGG  ACCCTACTCCCTCAATTTGG  TCACAGATCTCAAAGGCCAAGCGCATATCG  ACACCATGGCCAGTGGCGAAGTCATGATC  CACGCATGCCTTACTTACTTGTCATCGTCGTCCTTGTAGTCTCTAGACACCTTCTCTAATCTCG  GTCTTCGTATGTCGCCTTGG  ggccacgcgtcgactagtacnnnnnnnnnngatat  ggccacgcgtcgactagtac  ggccacgcgtcgactagtacnnnnnnnnnnacgcc  gtcatcgtcatccttgtaatcg  aaaTGATCACGAGATAGGGTTGAGTgTTG |
